# Two-stage, school and community-based population screening successfully identifies individuals and families at high-risk for type 2 diabetes: the Feel4Diabetes-study

**DOI:** 10.1186/s12902-019-0478-9

**Published:** 2020-03-12

**Authors:** Yannis Manios, Christina Mavrogianni, Christina-Paulina Lambrinou, Greet Cardon, Jaana Lindström, Violeta Iotova, Tsvetalina Tankova, Fernando Civeira, Jemina Kivelä, Zoltán Jancsó, Samyah Shadid, Kaloyan Tsochev, Rocío Mateo-Gallego, Sándorné Radó, George Dafoulas, Konstantinos Makrilakis, Odysseas Androutsos, on behalf of the Feel4Diabetes-study group, Yannis Manios, Greet Cardon, Jaana Lindström, Peter Schwarz, Konstantinos Makrilakis, Lieven Annemans, Ignacio Garamendi, Meropi Kontogianni, Odysseas Androutsos, Christina Mavrogianni, Konstantina Tsoutsoulopoulou, Christina Katsarou, Eva Karaglani, Irini Qira, Efstathios Skoufas, Konstantina Maragkopoulou, Antigone Tsiafitsa, Irini Sotiropoulou, Michalis Tsolakos, Effie Argyri, Mary Nikolaou, Eleni-Anna Vampouli, Christina Filippou, Katerina Gatsiou, Efstratios Dimitriadis, Tiina Laatikainen, Katja Wikström, Jemina Kivelä, Päivi Valve, Esko Levälahti, Eeva Virtanen, Vicky Van Stappen, Nele Huys, Ruben Willems, Samyah Shadid, Ivonne Panchyrz, Maxi Holland, Patrick Timpel, Stavros Liatis, George Dafoulas, Christina-Paulina Lambrinou, Angeliki Giannopoulou, Lydia Tsirigoti, Evi Fappa, Costas Anastasiou, Konstantina Zachari, Lala Rabemananjara, Maria Stella de Sabata, Winne Ko, Luis Moreno, Fernando Civeira, Gloria Bueno, Pilar De Miguel-Etayo, Esther Mª. Gonzalez-Gil, Maria I. Mesana, Germán Vicente-Rodriguez, Gerardo Rodriguez, Lucia Baila-Rueda, Ana Cenarro, Estíbaliz Jarauta, Rocío Mateo-Gallego, Violeta Iotova, Tsvetalina Tankova, Natalia Usheva, Kaloyan Tsochev, Nevena Chakarova, Sonya Galcheva, Rumyana Dimova, Yana Bocheva, Zhaneta Radkova, Vanya Marinova, Yuliya Bazdarska, Tanya Stefanova, Imre Rurik, Timea Ungvari, Zoltán Jancsó, Anna Nánási, László Kolozsvári, Csilla Semánova, Remberto Martinez, Marcos Tong, Kaisla Joutsenniemi, Katrina Wendel-Mitoraj

**Affiliations:** 1 Department of Nutrition and Dietetics, School of Health Science and Education, Harokopio University, 70 El Venizelou Ave, 176 71 Kallithea, Athens, Greece; 2 Department of Movement and Sports Sciences, Faculty of medicine and Health Sciences, Ghent University, Ghent, Belgium; 3 Department of Public Health Solutions, National Institute for Health and Welfare, Helsinki, Finland; 4 Department of Paediatrics, Medical University Varna, Varna, Bulgaria; 5 Department of Diabetology, Clinical Center of Endocrinology, Medical University Sofia, Sofia, Bulgaria; 6 Growth, Exercise, Nutrition and Development Research Group, School of Health Science, University of Zaragoza, Zaragoza, Spain; 7 Department of Family and Occupational Medicine, University of Debrecen, Debrecen, Hungary; 8 Department of Endocrinology, Ghent University Hospital, Ghent, Belgium; 9 Instituto de Investigación Sanitaria Aragón (IIS Aragón), CIBERCV, Zaragoza, Spain; 10 National and Kapodistrian University of Athens, Athens, Greece; 11 Department of Nutrition and Dietetics, School of Physical Education, Sport Science and Dietetics, University of Thessaly, Trikala, Greece

**Keywords:** Screening, School-based, Vulnerable groups, Type 2 diabetes, Hypertension

## Abstract

**Background:**

The implementation of population screening and early prevention strategies targeting individuals at high-risk for type 2 diabetes (T2D) seems to be a public health priority. The current work aimed to describe the screening procedure applied in the Feel4Diabetes-study and examine its effectiveness in identifying individuals and families at high risk, primarily for T2D and secondarily for hypertension, among vulnerable populations in low to middle-income countries (LMICs) and high-income countries (HICs) across Europe.

**Methods:**

A two-stage screening procedure, using primary schools as the entry-point to the community, was applied in low socioeconomic status (SES) regions in LMICs (Bulgaria-Hungary), HICs (Belgium-Finland) and HICs under austerity measures (Greece-Spain). During the first-stage screening via the school-setting, a total of 20,501 parents (mothers and/or fathers) of schoolchildren from 11,396 families completed the Finnish Diabetes Risk Score (FINDRISC) questionnaire, while their children underwent anthropometric measurements in the school setting. Parents from the identified “high-risk families” (*n* = 4484) were invited to participate in the second-stage screening, including the measurement of fasting plasma glucose (FPG) and blood pressure (BP). In total, 3153 parents participated in the second-stage screening (mean age 41.1 ± 5.6 years, 65.8% females).

**Results:**

Among parents who attended the second-stage screening, the prevalence of prediabetes (as defined by impaired fasting glucose; FPG 100-125 mg/dl) and T2D (FPG > 126 mg/dl) was 23.2 and 3.0% respectively, and it was found to be higher in the higher FINDRISC categories. The percentage of undiagnosed T2D among the participants identified with T2D was 53.5%. The prevalence of high normal BP (systolic BP 130-139 mmHg and/ or diastolic BP 85-89 mmHg) and hypertension (systolic BP ≥ 140 mmHg and/ or diastolic BP ≥ 90 mmHg) was 14 and 18.6% respectively, which was also higher in the higher FINDRISC categories. The percentage of cases not receiving antihypertensive treatment among the participants identified with hypertension was 80.3%.

**Conclusion:**

The findings of the current study indicate that the two-stage school and community-based screening procedure followed, effectively identified high-risk individuals and families in vulnerable populations across Europe. This approach could be potentially scalable and sustainable and support initiatives for the early prevention of T2D and hypertension.

**Trial registration:**

The Feel4Diabetes-intervention is registered at https://clinicaltrials.gov/ (NCT02393872; date of trial registration: March 20, 2015).

## Background

Diabetes affects around 8.8% of the adult population worldwide, and the total number of cases is predicted to rise from 425 million in 2017 to 629 million in 2045 [1]. T2D accounts for about 90% of all cases of diabetes and it is an important cause of mortality and morbidity, with its complications imposing a great burden both on individuals and healthcare systems [1, 2]. Furthermore, there is an elevated risk of developing T2D in vulnerable populations with disadvantaged socioeconomic background, probably due to the higher prevalence of unhealthy lifestyles in deprived communities and the barriers to access health care [3–5]. Based on evidence from several clinical trials, T2D is preventable through lifestyle intervention provided to individuals at increased risk [6, 7], while early detection of T2D may prevent mortality and complications [8, 9]. Thus, the implementation of screening strategies for the identification of high-risk individuals, coupled with non-pharmacological interventions becomes a public health priority.

The modifiable risk factors related to T2D (e.g. overweight/ obesity, physical inactivity, unhealthy dietary habits) that are developed in childhood and track into adulthood [10, 11], potentially increase the future T2D risk. Furthermore, these risk factors tend to cluster within a family, as its members share common genetic background, but also lifestyle, social and physical environment. In this context, interventions targeting families could be potentially more effective since the whole family becomes more supportive in adopting the desired behavior changes [12, 13]. Therefore, trying to identify families at high risk for developing T2D and invite them to take part in prevention programs could be a promising and potentially cost-effective approach, since at least two generations (i.e. parents and children) can be targeted simultaneously.

Using a two-stage screening procedure to identify individuals having or being at high risk of developing T2D is an easy to apply and cost-effective approach [14]. Furthermore, this approach seems to have an increased response rate compared to other multi-stage screening strategies [15] and is proposed by the guidelines of several international health organizations [16, 17]. During the first stage, a non-invasive assessment of risk with a self-reported diabetes risk evaluation tool is applied. Such a tool is the FINDRISC [18], which is the most widely used tool in Europe for estimating the future T2D risk. In the second stage, a diagnostic test (fasting glucose, oral glucose tolerance test [OGTT] or glycated hemoglobin [HbA1c]) is performed, but only among those individuals categorized as high-risk in the previous stage.

Although the two-stage screening has been used before in previous studies, in the vast majority of them its implementation was opportunistic, including individuals visiting healthcare services or applying door-to-door procedures [15]. However, this approach seems to be less effective in reaching and screening less health-conscious individuals, early middle-aged adults and vulnerable population groups, such as low SES groups [19]. Therefore if we want to reach such populations, an alternative systematic, low-cost and potentially sustainable approach needs to be considered. School setting could facilitate this need and serve as the entry-point to the community, in order to identify “high-risk families”.

Considering the above, the Feel4Diabetes consortium, developed and implemented for the first time a two-stage screening procedure targeting vulnerable families in six European countries. Primary schools were used as the entry-point to the community, aiming to reach and screen the targeted population (children and their families). The primary aim of the current work was to describe the procedure of the Feel4Diabetes-screening and present the relevant results in terms of identifying individuals and families at high risk for T2D. Furthermore, since T2D and hypertension share common comorbidities and risk factors [20], the secondary aim of the current work was to examine whether the screening procedure followed could identify also individuals and families at high risk for hypertension.

## Methods

### Study background

The current work used the baseline data of the EU-funded Feel4Diabetes-study (http://feel4diabetes-study.eu/), which aimed to develop, implement and evaluate a school- and community-based intervention to prevent T2D among families from vulnerable groups across Europe. A detailed description of the study design has been previously published [21]. Recruitment started in January 2016 and measurements were conducted between April and June in Belgium, Greece and Spain, while for Finland, Hungary and Bulgaria measurements were extended to September 2016. The Feel4Diabetes-study was registered at clinicaltrials.gov as NCT02393872.

### Recruitment

Recruitment was based on a standardized, multi-stage sampling procedure and was conducted within selected provinces in six European countries. The participating countries are categorized in three socioeconomic levels according to the World Bank country classification based on Gross National Income (GNI) per capita [22] and the Eurostat’s Government Budget Deficit data in 2014 [23]. Specifically the participating countries were grouped as follows: LMICs (i.e. Bulgaria and Hungary), HICs (i.e. Belgium and Finland) and HICs under austerity measures (i.e. Greece and Spain). Since the prevalence of T2D tends to be higher in LMICs [24], any municipality, school district or other equivalent unit in Bulgaria and Hungary was considered as “vulnerable” area and eligible to participate in the study. Regarding HICs, as low SES has been associated with increased risk of T2D in these countries [3], only low SES regions were considered as “vulnerable” in Belgium, Finland, Greece and Spain. Specifically, in these countries low SES regions were defined using the following steps: (i) the municipalities, school districts or other equivalent units in the selected provinces were grouped in tertiles according to SES indices (i.e. literacy or unemployment rates) retrieved from official resources and relevant authorities [25–28] and (ii) “vulnerable” areas were randomly selected only from the tertile with the lowest SES indices.

In each country, primary schools located in the selected “vulnerable” areas were used as the entry-point to the community. Children attending the first three grades of compulsory education as well as their parents and grandparents (wherever feasible) were recruited to the study. Of these recruited families, the “high-risk families” were identified based on T2D risk estimation, using the FINDRISC questionnaire. A family was regarded as “high-risk” if at least one parent fulfilled the country-specific cut-off point for FINDRISC that indicated increased T2D risk (for the majority of countries, considering the young age of the participants, that was set as a FINDRISC score ≥ 9).

### First-stage screening

In the participating schools, teachers distributed the standardized study questionnaire to all children in the class to be delivered to their parents/ caregivers [29]. Families were requested to return the completed questionnaires in sealed envelopes, and the children from the participating families underwent anthropometric measurements at school, conducted by trained research assistants [30].

### Measurements conducted during the first-stage screening

#### Questionnaires

Data regarding children’s and parents’ eating, physical activity and sedentary behaviors, and parents’ sociodemographic data were collected, while both parents were requested to complete the FINDRISC questionnaire. The most recent version of it consists of eight scored questions that cover the well-known risk factors of T2D, i.e. age, body mass index (BMI), waist circumference (WC), daily physical activity, daily consumption of vegetables and fruits/ berries, use of antihypertensive medication, individual’s history of high blood glucose, and family history of diabetes [31]. The total score indicates the individual’s 10-year risk of developing T2D and ranges from 0 to 26, as follows: < 7 (low), 7–11 (slightly elevated), 12–14 (moderate), 15–20 (high) and > 20 (very high). In order to facilitate the assessment of WC, a paper waist measuring tape was provided to each family.

#### Anthropometry

Body weight was measured to the nearest 0.1 kg and children were weighed in light clothing and without shoes. Height was measured to the nearest 0.1 cm with the children standing barefoot, keeping shoulders in a relaxed position, arms hanging freely and head in Frankfurt horizontal plane. Two readings were obtained out of each measurement and the mean was used for the analysis. A third measurement was conducted if the previous measurements differ > 100 g for weight and > 1 cm for height. Weight and height were used to calculate BMI using Quetelet’s equation [weight (kg)/height (m)^2^]. The International Obesity Task Force (IOTF) cutoff points [32] were used to categorize children as “underweight”, “normal weight”, “overweight”, or “obese”.

### Second-stage screening

Following the first-stage screening and after calculating the FINDRISC scores and identifying the “high-risk families”, researchers contacted these families in a discrete manner (to avoid stigmatization of children/ families) and invited them to attend the second-stage screening, which was conducted outside the school setting. Specifically, all parents and grandparents (wherever feasible) of the identified “high-risk families” were invited to undergo a brief medical check-up delivered in local community centers or during home visits (in certain cases in Belgium). In all countries, measurements were conducted by trained research assistants [30], using standardized protocols and calibrated equipment. The current work focuses on the early identification of T2D and hypertension among parents (early middle-aged adults) and therefore grandparents’ data were excluded from the statistical analysis and relevant tables.

### Measurements conducted during the second-stage screening

#### Blood indices

Blood samples were drawn in the morning after overnight fasting for at least 8 h. Participants (parents and grandparents where feasible) were contacted via phone calls on the previous day to ensure compliance with fasting. Moreover, they were advised to refrain from taking their medications in the morning when the blood collection was performed. Fasting plasma glucose (FPG) was analyzed in accredited laboratories, using similar enzymatic assays in all study centers. Participants were classified according to the American Diabetes Association (ADA) criteria [33] in the following categories: normal (FPG < 100 mg/dl; 5.6 mmol/l), prediabetes (FPG 100-125 mg/dl; 5.6–6.9 mmol/l) and T2D (FPG ≥ 126 mg/dl; 7.0 mmol/l).

#### Blood pressure measurement

Blood pressure (BP) was measured on the right arm, in a sitting position using an Omron digital blood pressure measuring device (OMRON M6 or M6 AC) after 5 min of rest, on three occasions, allowing 1 min interval among the occasions. Participants were asked to avoid vigorous exercise, smoking, eating or drinking for 1 h before the BP measurement. Means of the second and third measurements were used in the current statistical analysis. Participants were classified according to the European guidelines [34] in the following categories: optimal or normal (systolic BP < 130 mmHg and/ or diastolic BP < 85 mmHg), high normal (systolic BP 130-139 mmHg and/ or diastolic BP 85-89 mmHg) and hypertension (systolic BP ≥ 140 mmHg and/ or diastolic BP ≥ 90 mmHg), with the BP category to be defined by the highest level of BP, either systolic or diastolic.

#### Anthropometry

Body weight and height were measured in light clothing and without shoes to the nearest 0.1 kg and 0.1 cm respectively, and WC was measured midway between the lowest rib margin and the iliac crest to the nearest 0.1 cm. Portable equipment was used [for weight: digital scales (SECA 813 or 877), for height: telescopic stadiometers (SECA 213 or 214 or 217 or 225), for WC: non-elastic tapes (SECA 201)]. Two readings were obtained out of each measurement and the mean was used for the analysis. A third measurement was conducted if the previous measurements differ > 100 g for weight, > 1 cm for height and > 1 cm for WC. BMI and WC were classified based on the World Health Organization (WHO) criteria [35].

#### Questionnaires

Besides data related to lifestyle factors, sociodemographic data (e.g. age, gender, education, occupation) and information regarding medical history and medication use were collected from participants (parents and grandparents where feasible). Specifically, previous diabetes diagnosis was assessed by the question “Has a physician ever told you that you have diabetes?”, while medication-treated hypertension was assessed by the question “Do you currently use drugs for high BP prescribed by a physician?” (Possible answers: yes, no).

### Statistical analysis

In the current work the participating countries were grouped in regions as: HICs (Belgium and Finland), HICs under austerity measures (Greece and Spain) and LMICs (Bulgaria and Hungary). A descriptive statistical analysis was performed by using the Statistical Package for Social Sciences (SPSS Inc., Chicago, IL, USA), version 21.0. Continuous variables are presented as means ± standard deviations and categorical values as proportions (%). Differences in continuous variables were assessed by parametric (One-Way Analysis of Variance) or nonparametric tests (Kruskall-Wallis), according to the distribution of the variables (Kolmogorov-Smirnoff test), while Pearson’s Chi-square test was used to evaluate the differences in proportions. All statistical tests were two-tailed and the level of statistical significance was set at *P* < 0.05.

## Results

The population reached in the Feel4Diabetes-study is presented in Table 1. Overall, 28,075 families were contacted via the participating 219 primary schools. During the first-stage screening that was delivered via the school setting with the completion of a self-reported screening tool (i.e. FINDRISC), 11,396 families were screened for T2D risk. All parents and/or grandparents of those families identified with increased risk were invited to the second-stage screening (a more detailed medical examination) and finally at least one adult from 2537 “high-risk families” was measured at baseline, with data available for 3153 parents.

**Table 1:** Population reached and screened in the “Feel4Diabetes-study”

Number of	All countries	HICs under austerity measures		LMICs		HICs	
		Greece	Spain	Bulgaria	Hungary	Belgium	Finland
Participating schools	219	56	41	20	14	58	30
Families contacted via participating schools	28,075	5195	4823	6541	2902	5367	3247
Families participated in the “first-stage screening”	11,396	2096	1567	2948	1762	1684	1339
Parents provided completed FINDRISC questionnaires (“first-stage screening”)	20,501	3741	3043	5211	3034	2990	2482
Children measured at school setting (anthropometric indices obtained)	12,194	2286	1703	3034	1867	1798	1506
Families identified as “high risk families” and invited to participate in the “second-stage screening”	4484	907	715	1088	689	475	610
Families participated in the “second-stage screening”	2537	452	541	463	286	420	375
Parents participated in the “second-stage screening”	3153	696	659	554	319	499	426
Grandparents participated in the “second-stage screening”	121	69	–	46	1	5	–

*FINDRISC* Finnish Diabetes Risk Score, *HICs* High-income countries, *LMICs* Low to middle-income countries

The descriptive characteristics of the parents and children of the “high-risk families” that participated in the second-stage screening, stratified by region are presented in Table 2. Parents were aged 41.1 ± 5.6 years and 65.8% were females. Overall, 65.8% of parents were full-time employed; while 23.6% of them had less than 12 years of education, with the latter being lower in HICs. The prevalence of overweight and obesity was 35.3 and 36.7%, respectively, in the total sample and it was found to be lower in LMICs compared to other regions. Finally, among the participating “high-risk families”, 2711 children (aged 8.16 ± 1.0 years; 51.1% girls) were measured.

**Table 2:** Descriptive characteristics of parents and children of families participated in the second-stage screening by region

	Total sample	HICs under austerity measures	LMICs	HICs	*P-*value*
Parents	(*n* = 2849)	(*n* = 1385)	(*n* = 624)	(*n* = 840)	
Age (years)	41.1 (5.56)	42.4 (5.32)^a,b^	39.6 (5.40)^a^	40.1 (5.60)^b^	< 0.001
Females, n (%)	1875 (65.8)	837 (60.4)^a,b^	464 (74.4)^a^	574 (68.3)^b^	< 0.001
Occupation, n (%)					
Work full- time	1875 (65.8)	885 (63.9)^a^	445 (71.3)^a,b^	545 (64.8)^b^	< 0.001
Work part- time	335 (11.7)	154 (11.1)^a,b^	41 (6.5)^b,c^	140 (16.7)^a,c^	
Stay at home parent	345 (12.1)	203 (14.7)^a^	83 (13.3)^b^	59 (7.0)^a,b^	
Unemployed	139 (4.9)	112 (8.1)^a,b^	8 (1.3)^a^	19 (2.3)^b^	
Full-time education	31 (1.1)	4 (0.3)^a,b^	8 (1.3)^a^	19 (2.3)^b^	
Other**	124 (4.4)	27 (1.9)^a,b^	39 (6.3)^a^	58 (6.9)^b^	
Educational level, n (%)					
< 12 years	673 (23.6)	373 (26.9)^a^	160 (25.6)^b^	140 (16.7)^a,b^	< 0.001
≥ 12 years	2176 (76.4)	1012 (73.1)^a^	464 (74.4)^b^	700 (83.3)^a,b^	
Weight status, n (%)					
Overweight	1006 (35.3)	506 (36.5)^a^	185 (29.6)^a,b^	315 (37.5)^b^	< 0.001
Obese	1045 (36.7)	550 (39.7)^a^	203 (32.6)^a^	292 (34.8)	
Children	(*n* = 2711)	(*n* = 1089)	(*n* = 753)	(*n* = 869)	
Age (years)	8.16 (1.02)	7.80 (0.93)^a,b^	8.43 (1.00)^a^	8.36 (1.00)^b^	< 0.001
Females, n (%)	1385 (51.1)	549 (50.4)	393 (52.2)	443 (51.0)	0.752

Data are means (SD) except where noted otherwise

^*^P-values indicate the significance of the differences among regions

Figures sharing the same superscript letters differentiate significantly from each other

^**^Other includes “retired” and “something else”

*HICs* High-income countries, *LMICs* Low to middle-income countries

**Fig. 1:**
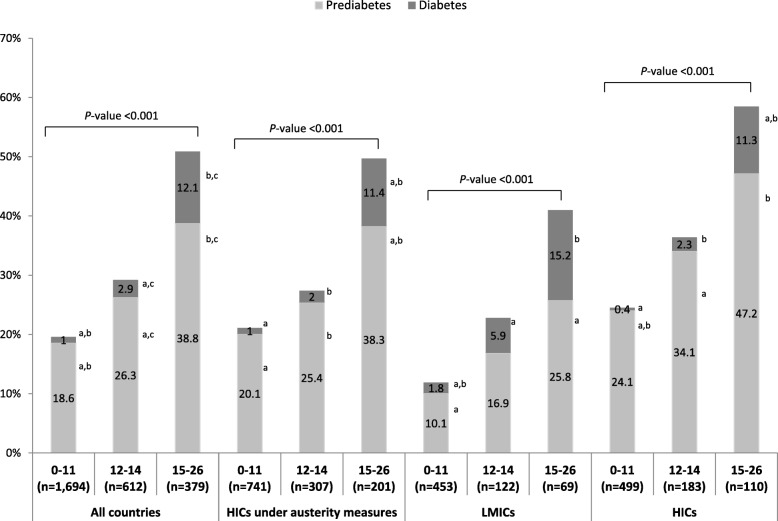
Prevalence of prediabetes and diabetes in parents participated in the second-stage screening by FINDRISC category. *P*-values indicate the significance of the differences among FINDRISC categories: 0–11 (low and slightly elevated risk), 12–14 (moderate risk), 15–16 (high and very high risk). Figures sharing the same superscript letters differentiate significantly from each other. *FINDRISC:* Finnish Diabetes Risk Score; *HICs*: High-income countries; *LMICs*: Low to middle-income countries

The prevalence of prediabetes and T2D among the parents that participated in the second-stage screening is presented in Table 3. According to the measured FPG and the ADA criteria, the overall prevalence of prediabetes was 23.2%, while for T2D it was 3.0%. A marked higher prevalence of prediabetes and T2D was observed in parents with higher FINDRISC (*P* < 0.001) in all countries, as well as in each region (Fig. 1). Overall, among those parents identified with T2D, 53.5% of participants were previously undetected, with this percentage being lower in the highest FINDRISC category compared to the lowest one (*P* = 0.007).

**Table 3:** Prevalence of prediabetes and diabetes in parents participated in the second-stage screening

	I	II	III	IVParticipants as presented in column III, stratified by FINDRISC category			*P*-value*
	Participants identified with prediabetes, n (%)	Participants identified with diabetes, n (%)	Participants for whom diabetes was previously undetected among those identified with diabetes in column II, n (%)**				
				0–11	12–14	15–26	
All countries (*n* = 2685)	623 (23.2)	81 (3.0)	40 (53.5)	13 (81.3)^a^	13 (64.7)	14 (36.8)^a^	0.007
HICs under austerity measures (*n* = 1249)	304 (24.3)	36 (2.9)	15 (40.0)	4 (57.1)	5 (66.7)	6 (27.3)	0.127
LMICs (*n* = 644)	84 (13.1)	26 (4.0)	21 (80.0)	8 (100.0)	7 (85.7)	6 (60.0)	0.098
HICs (*n* = 792)	235 (29.6)	19 (2.4)	4 (36.4)	1 (100.0)	1 (25.0)	2 (33.3)	0.368

^*^P-values indicate the significance of the differences among FINDRISC categories

Figures sharing the same superscript letters differentiate significantly from each other

^**^ The numbers provided for this variable in the case of HICs do not include data from Finland (*n* = 7), since participants with previously diagnosed diabetes were excluded from the study at this study center

*FINDRISC* Finnish Diabetes Risk Score, *HICs* High-income countries, *LMICs* Low to middle-income countries

**Fig. 2:**
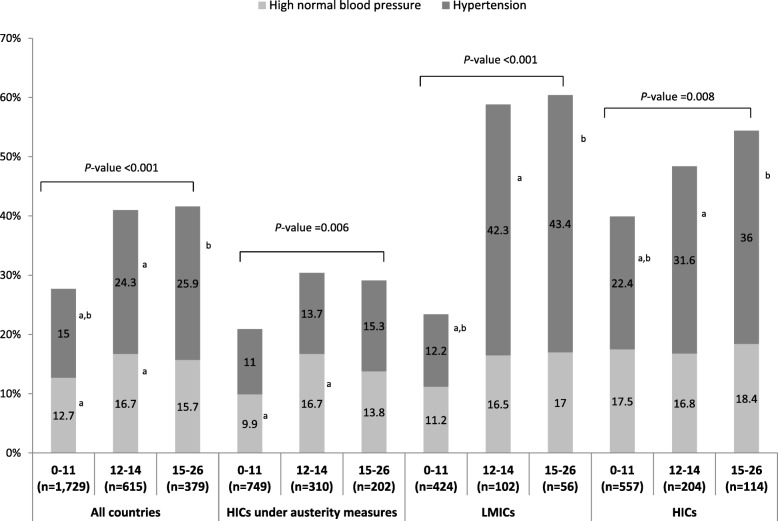
Prevalence of high normal blood pressure and hypertension in parents participated in the second-stage screening by FINDRISC category. *P*-values indicate the significance of the differences among FINDRISC categories: 0–11 (low and slightly elevated risk), 12–14 (moderate risk), 15–16 (high and very high risk). Figures sharing the same superscript letters differentiate significantly from each other. *FINDRISC:* Finnish Diabetes Risk Score; *HICs:* High-income countries; *LMICs:* Low to middle-income countries

The prevalence of high normal BP and hypertension among parents that participated in the second-stage screening are presented in Table 4. The overall prevalence of high normal BP was 14.0%, ranging from 12.7 and 16.7% to 15.7% for participants with FINDRISC score ≤ 11, 12–14 and ≥ 15, respectively (Fig. 2). The prevalence of hypertension was 18.6% in the total sample, while 80.3% of the participants identified with hypertension were not receiving any antihypertensive treatment. The prevalence of hypertension was found to be higher in the higher FINDRISC categories compared to the lowest one (Fig. 2), while the percentage of participants not receiving any antihypertensive treatment among those identified with hypertension was found to be lower in the higher FINDRISC categories compared to the lowest ones (*P* < 0.001).

**Table 4:** Prevalence of high normal blood pressure and hypertension in parents participated in the second-stage screening

	I	II	III	IVParticipants as presented in column III, stratified by FINDRISC category			*P*-value*
	Participants identified with high normal blood pressure, n (%)	Participants identified with hypertension, n (%)	Participants not receiving antihypertensive treatment among those identified with hypertension, n (%)				
				0–11	12–14	15–26	
All countries (*n* = 2723)	382 (14.0)	507 (18.6)	407 (80.3)	231 (89.2)^a,b^	116 (77.1)^a,c^	60 (61.7)^b,c^	< 0.001
HICs under austerity measures (n = 1261)	154 (12.2)	156 (12.4)	121 (77.5)	74 (88.8)^a^	31 (73.2)	16 (53.3)^a^	< 0.001
LMICs (*n* = 582)	74 (12.7)	119 (20.5)	84 (70.8)	41 (79.6)	29 (68.3)	14 (56.5)	0.121
HICs (*n* = 880)	154 (17.5)	232 (26.4)	202 (87.0)	117 (93.3)^a^	55 (85.5)	30 (70.7)^a^	0.001

*P-values indicate the significance of the differences among FINDRISC categories

Figures sharing the same superscript letters differentiate significantly from each other

*FINDRISC* Finnish Diabetes Risk Score, *HICs* High-income countries, *LMICs* Low to middle-income countries

The prevalence of overweight or obesity in the children of “high-risk families” participating in the second-stage screening is presented in Table 5. This was found to be 28.2% in the total sample, with this percentage being higher if the parents were at higher T2D risk (i.e. at least one parent with FINDRISC score 12–14 or ≥ 15 vs. both parents with FINDRISC score ≤ 11, P < 0.001). The same trend was also observed for HICs under austerity measures, while no significant differences were found among parental FINDRISC categories for the other regions.

**Table 5:** Prevalence of overweight or obesity in children of families participated in the second-stage screening

Variables	Total sample	By parental FINDRISC category			
		Both parents 0–11	At least one parent 12–14	At least one parent 15–26	*P*-value*
Overweight or obesity in children, n (%)					
All countries (*n* = 2231)	630 (28.2)	267 (24.5)^a,b^	213 (31.4)^a^	150 (32.5)^b^	0.001
HICs under austerity measures (*n* = 930)	309 (33.2)	126 (28.9)^a^	100 (35.1)	83 (39.7)^a^	0.018
LMICs (*n* = 616)	175 (28.4)	73 (24.6)	64 (32.7)	38 (30.9)	0.119
HICs (*n* = 685)	146 (21.3)	68 (19.0)	49 (24.9)	29 (22.3)	0.258

*P-values indicate the significance of the differences among parental FINDRISC categories

Figures sharing the same superscript letters differentiate significantly from each other

*FINDRISC* Finnish Diabetes Risk Score, *HICs* High-income countries, *LMICs* Low to middle-income countries

## Discussion

In the Feel4Diabetes-study, a large number of families were screened and a relatively high number of parents were identified to be at high risk for T2D and hypertension via a two-stage screening procedure, trying to keep the cost as low as possible. That was realized via the use of available community infrastructure (primary schools and local municipality centers) and personnel (i.e. teachers and healthcare professionals wherever feasible). The current approach, using the school-setting as the entry-point to the community, can be a potentially cost-effective and sustainable community-based approach for the early identification of high-risk individuals and families for T2D and hypertension and support relevant prevention initiatives. Overall, the implemented screening procedure as well as the definition and prioritization of vulnerable population-groups, could guide other similar strategies both in LMICs and HICs across Europe.

Considering the relatively young age (41.1 ± 5.6 years) of the study population (i.e. parents of primary-school children), recruited in countries across Europe, the fact that about only 66% of them reported to be working full-time and almost 25% had less than 12 years of education, indicate that the aim of the Feel4Diabetes-study to target primarily vulnerable groups was achieved to a large extent. Selecting the most appropriate screening strategy to reach such a population (i.e. early middle-aged adults and populations in low SES areas) poses challenges for healthcare professionals. Although opportunistic screening for T2D among asymptomatic individuals has been recommended as the most efficient approach [36], this may lead to selection bias due to reaching the most motivated individuals. On the other hand, the door-to-door screening approach can target a large segment of the population, but it has been reported to have lower adherence to the diagnostic tests compared to opportunistic screening [37] and may not be a sustainable and easily scalable approach. To overcome these issues, the findings of the current study indicate that applying a two-stage screening using primary schools as the entry-point to the community can efficiently reach high-risk families/ individuals. Therefore, by embedding this procedure into the local or national school and local healthcare systems, a systematic, continuous and organized screening could be delivered at population level and potentially reach the largest part of the community.

The screening approach used in the Feel4Diabetes-study managed to identify 3.0 and 23.2% of individuals with T2D and prediabetes, respectively, and these percentages were found to be increased in the higher FINDRISC categories. These figures seem to be lower compared to findings from some other studies [38, 39], possibly due to the screening approach used in the current study (i.e. targeting the whole population and not only the health conscious ones or those attending healthcare services for other health issues), as well as the younger age of the participants. Furthermore, 53.5% of cases identified with T2D were previously undetected. This is in accordance with current estimations indicating that almost half of all people with diabetes are undiagnosed [1]. It has been also reported that the FINDRISC questionnaire is considered valid for identifying individuals with undiagnosed T2D and dysglycaemia in the population under study, while “individuals’ history of high blood glucose” was the FINDRISC component most strongly associated with both conditions [40]. In this context, the proportion of undetected T2D cases was found to be highest in the lowest FINDRISC category, probably because these individuals obtained a low score in the aforementioned FINDRISC component. To some extent this can be attributed to the fact that many of these participants may have not previously undergone any glycaemia testing, most likely because of their relatively young age, lack of other risk factors or lack of awareness [41].

Prevalence of high BP was comparable with other studies conducted in European countries [42–44], although with large variations among countries, sexes or SES groups (relevant data from the Feel4Diabetes-study are not presented in the current paper). In correspondence with the prevalence of T2D and prediabetes by FINDRISC category observed in the current study, a similar trend was also reported for hypertension and high normal BP, with the prevalence being higher in the higher FINDRISC categories. Indeed, data from a prospective study has shown that FINDRISC can also identify subjects at high risk for developing hypertension in the long-term [45]. Furthermore, in the Feel4Diabetes-study a high proportion (≈80.0%) of the individuals identified with hypertension was not receiving any antihypertensive treatment. Similar high percentages have been previously reported from population studies [46]. Based on available literature [46, 47], probably many individuals were not aware of their condition, but this can only be speculated in the current study, since the awareness of having hypertension/ high BP was not assessed. Last but not least, individuals that had taken medications for high blood pressure on a regular basis at some point in their life, scored higher in the FINDRISC. This could probably explain why the proportion of undetected cases of hypertension among the individuals identified with hypertension was lower in the highest FINDRISC category.

Finally, about 30% of the children from the families participating in the second-stage screening were overweight or obese, with this percentage being higher in families that at least one parent was at higher-risk for T2D based on self-reported FINDRISC data. Overall, more than 70% of the parents attending the second-stage screening were found to be overweight or obese. Given the positive association between parental and children’s weight status [48], the implementation of a lifestyle intervention targeting the whole family could have multiple benefits for both high-risk parents and their offspring. Emphasizing to parents that serve as role models for their children [49], could further motivate them to apply lifestyle changes for their own benefit, as well as for the benefit of their children.

## Conclusions

The findings of the current work demonstrate that the systematic, two-stage screening procedure implemented in the Feel4Diabetes-study using the school as the entry-point to the community can identify individuals and families at high risk for T2D and /or hypertension. Furthermore, considering the relatively young age of the adult participants/parents in the current study, a large proportion of undetected T2D and/or hypertension was also identified. As the current approach is easy-to-apply and with relatively low cost, it could be considered potentially scalable and sustainable for the early identification of high-risk individuals and families (also within vulnerable population-groups), and support initiatives for the prevention of T2D and hypertension.

## Data Availability

The datasets generated and/or analysed during the current study are not publicly available, since the data used is confidential based on Feel4Diabetes publications rules, but are available from the corresponding author on reasonable request.

## Abbreviations

BMI: Body Mass Index
BP: Blood Pressure
FINDRISC: Finnish Diabetes Risk Score
FPG: Fasting plasma glucose
HICs: High-income countries
LMICs: Low to middle-income countries
T2D: Type 2 Diabetes
